# A Novel Attenuated NADC30‐Like PRRSV Vaccine Candidate Provides Cross‐Protection Against Homologous and Heterologous Viral Challenge

**DOI:** 10.1155/tbed/5955745

**Published:** 2026-07-05

**Authors:** Heng Zhang, Jiakai Zhao, Chengxin Zhang, Ting Tang, Qianyi Zhu, Hui Zhou, Yingru Ma, Xiaohong Qi, Qin Zhao, Yani Sun

**Affiliations:** ^1^ College of Veterinary Medicine, Northwest A&F University, Yangling, 712100, Shaanxi, China, nwsuaf.edu.cn; ^2^ Swine Disease R&D Center, Shandong SINDER Technology Co.,Ltd., Qingdao, Shandong, 266104, China; ^3^ Engineering Research Center of Efficient New Vaccines for Animals, Ministry of Education, Yangling, Shaanxi, 712100, China, moe.edu.cn; ^4^ Key Laboratory of Ruminant Disease Prevention and Control (West), Ministry of Agriculture and Rural Affairs, Yangling, Shaanxi, 712100, China, agri.gov.cn

**Keywords:** attenuated, efficacy, live vaccine, NADC30-like, NSP2 dual-gene deletion, PRRSV, safety

## Abstract

Porcine reproductive and respiratory syndrome (PRRS) virus (PRRSV) is one of the major pathogens threatening the global swine industry and causes enormous economic losses annually. Vaccination remains one of the most important strategies for the prevention and control of PRRSV infection. In this study, a novel genotype 2 NADC30‐like PRRSV strain, designated AHFY, was isolated and characterized from eastern China. Phylogenetic analysis revealed that strain AHFY belonged to sublineage 1.8. An attenuated strain, AHFY‐F140, was obtained through 140 serial passages in MARC‐145 cells. Genomic sequence comparison between AHFY‐F140 and the parental strain AHFY‐F1 identified two discontinuous amino acid (aa) deletions in the nonstructural protein 2 (NSP2) gene, spanning residues 355–364 and 391–583, which were first detected at passage 90 and persisted through passage 140. The viral titer gradually increased with passaging, rising from 10^4.3^ TCID_50_/mL for the parental strain AHFY‐F1 to 10^8.7^ TCID_50_/mL for AHFY‐F140. A pathogenicity evaluation was conducted in 4‐week‐old PRRSV‐free New American Line piglets using AHFY‐F1 and AHFY‐F140. Piglets inoculated with AHFY‐F1 developed persistent fever and obvious respiratory clinical signs, accompanied by severe and typical histopathological lung lesions. In contrast, piglets inoculated with AHFY‐F140 showed no fever or other clinical signs. PRRSV‐specific antibodies became positive at 11 days postinoculation (dpi) in AHFY‐F140‐inoculated piglets, which was later than the 7 dpi observed in AHFY‐F1‐inoculated piglets. Furthermore, immunization with AHFY‐F140 provided robust protection against subsequent challenge with either the parental strain AHFY‐F1 or the highly pathogenic PRRSV (HP‐PRRSV) strain JXA1 (lineage 8). Collectively, these findings demonstrate that the attenuated strain AHFY‐F140 represents a promising vaccine candidate against both NADC30‐like and JXA1‐like PRRSV infections.

**Summary**

• A novel quadruple‐parent recombinant NADC30‐like porcine reproductive and respiratory syndrome (PRRS) virus (PRRSV) strain AHFY, featuring an NADC30 (lineage 1) backbone interspersed with genomic segments from CH‐1R (nucleotide [nt] 5399–8141), TJM‐F92 (nt 8710–8950), and QYYZ (nt 11,984–13,616), was isolated, and its attenuated derivative AHFY‐F140 was generated after 140 serial passages in MARC‐145 cells.
• The AHFY‐F140 strain harbors novel, stable large‐fragment dual deletions (amino acids [aas] 355–364 and 391–583) within the virulence‐associated region of nonstructural protein 2 (NSP2), alongside stable mutations in GP5.
• AHFY‐F140 provided effective protection against challenge with both the parental NADC30‐like strain AHFY‐F1 and the highly pathogenic PRRSV (HP‐PRRSV) strain JXA1, highlighting its potential as a broad‐spectrum vaccine candidate.

## 1. Introduction

Porcine reproductive and respiratory syndrome (PRRS), caused by the PRRS virus (PRRSV), is a globally endemic swine disease characterized by respiratory distress in piglets and reproductive failure in pregnant sows, inflicting substantial economic losses on the swine industry [1]. PRRSV is an enveloped, positive‐sense, single‐stranded RNA virus classified in the genus *Porartevirus*, family Arteriviridae, and order Nidovirales [2]. Its genome is ~15 kb in length and harbors 12 open reading frames (ORFs). The 5′‐terminal ORF1a and ORF1b encode polyproteins PP1a and PP1ab, which account for more than two‐thirds of the genome and are proteolytically cleaved into 16 nonstructural proteins (NSPs: NSP1α, NSP1β, NSP2, NSP2TF, NSP2N, NSP3‐6, NSP7α, NSP7β, and NSP8‐12) that are responsible for viral replication and transcription [2]. The remaining ORFs (ORF2‐7) encode structural proteins, including five minor envelope proteins (GP2a, E, GP3, GP4, and ORF5a), two major envelope proteins (GP5 and M), and the nucleocapsid protein (N) [3].

PRRSV comprises two species: Betaarterivirus suid 1 (PRRSV‐1) and Betaarterivirus suid 2 (PRRSV‐2). (ICTV, 2021), which share ~60% whole‐genome nucleotide (nt) identity [4]. Based on phylogenetic analysis of ORF5, PRRSV‐2 can be categorized into 11 major lineages [1].

First identified in North America in 1987, PRRSV rapidly disseminated to Europe and Asia [5]. In China, the disease was first reported in 1995 [6]. Notably, a highly pathogenic PRRSV (HP‐PRRSV) variant belonging to lineage 8 emerged in 2006, triggering severe outbreaks and becoming the dominant epidemic strain [7]. Concurrently, the NADC30 strain (lineage 1) was isolated in North America in 2008 [8]. Since 2013, NADC30‐like strains with high genetic homology to the original NADC30 strain have been detected in China and have subsequently become predominant in the field [9]. These strains are characterized by typical deletions in the NSP2 gene, including a 11‐amino‐acid (aa) deletion (positions 322–332), a 1‐aa deletion (position 483), and a 19‐aa deletion (positions 504–522) [10].

Vaccination remains the primary strategy for PRRSV control [11]. Modified live vaccines (MLV), generated via serial passaging in African green monkey kidney (MARC‐145) cells, have been widely adopted in the swine industry [12]. However, the rapid evolution and frequent recombination of field PRRSV strains limit the cross‐protective efficacy of MLV vaccines against heterologous strains [13]. For instance, although MLV vaccines targeting HP‐PRRSV were developed following the 2006 outbreak, their efficacy against emerging NADC30‐like strains has proven insufficient, with vaccine failure reported in multiple Chinese provinces [14, 15].

The limited cross‐protection between lineage 8‐derived MLVs and lineage 1 NADC30‐like strains is largely attributable to extensive genetic divergence, particularly within NSP2—the most variable region of the PRRSV genome. Accumulating evidence has established that NSP2 plays multifaceted roles in viral virulence, cell tropism, and vaccine attenuation. For instance, the full‐length NSP2 of HP‐PRRSV‐2 acts as a bridging molecule that facilitates the assembly of the nucleocapsid protein with viral envelope proteins, a function essential for productive virion formation [16]. Conversely, NSP2 is also a key determinant of viral uncoating in primary porcine alveolar macrophages (PAMs); a vaccine‐type nsp2 substitution leads to a replication defect at the uncoating stage, thereby attenuating virulence [17]. Moreover, the hypervariable region of NSP2 (aa 323–521) directly governs PRRSV tropism for PAMs, and deletion of this region abolishes viral replication in PAMs and pathogenicity in pigs while retaining replication in MARC‐145 cells [18]. From an immunological perspective, HP‐PRRSV NSP2 acts as a potent virulence factor by inducing excessive inflammatory responses (e.g., upregulation of TLR4, IL‐1β, and MPO), whereas NSP2 from low‐virulence NADC30‐like strains elicits minimal immune activation, favoring immune evasion and viral persistence [19]. These findings collectively indicate that adaptive mutations, particularly large deletions in the NSP2 hypervariable region acquired during serial passage in the cell culture, represent a rational and common mechanism for PRRSV attenuation. Therefore, developing a lineage 1‐matched MLV vaccine with characteristic NSP2 deletions offers a promising strategy to overcome the cross‐protection barrier.

In the present study, we isolated a prevalent NADC30‐like PRRSV strain, attenuated it through serial passaging in MARC‐145 cells, and systematically analyzed its genomic characteristics and pathogenicity in piglets. Furthermore, this passaging‐attenuated strain was demonstrated to provide effective immunoprotection for piglets. These findings enhance our understanding of PRRSV evolutionary dynamics and offer a promising MLV vaccine candidate for the control of NADC30‐like PRRSV infections.

## 2. Materials and Methods

### 2.1. Virus and Cells

Primary PAMs were isolated from a 30‐day‐old PRRSV‐free piglet (Shandong Xincheng Animal Husbandry Technical Service Co., Ltd., China), as previously described [20]. Briefly, the lung was collected under sterile conditions, rinsed with phosphate‐buffered saline (PBS, 0.1 mol/L, pH 7.2), and gently tapped to collect the lavage fluid. After centrifugation at 300 × *g* for 10 min, the PAM pellet was resuspended in RPMI 1640 medium (Biological Industries, Israel) supplemented with 10% fetal bovine serum (FBS) and cultured at 37°C in a 5% CO_2_ incubator. MARC‐145 cells (approved by the Ethics Committee of Northwest A&F University, License Number 20220301) were used for virus propagation and titration. The monoclonal antibody (mAb) 6D10 was generated and maintained in our laboratory, as previously reported [21]. The JXA1 strain (GenBank Accession Number EF112445.1) was kindly provided by Professor Xiao Shuqi (Lanzhou Veterinary Research Institute, Chinese Academy of Agricultural Sciences).

### 2.2. Sample Collection and Virus Isolation

In 2022, an outbreak occurred in a large‐scale pig farm in Anhui Province, affecting sows and weaned piglets with ~20% morbidity and 10% mortality. Affected piglets exhibited listlessness, anorexia, high fever, cough, dyspnea, ear and extremity cyanosis, and bluish‐purple skin discoloration. Necropsy revealed pleural effusion, extensive fibrinous exudation on lung surfaces, and hyperemic, congested, and dark‐red lungs.

Viral nucleic acids were extracted from lung tissue samples. Multiplex quantitative real‐time PCR (qPCR/RT‐PCR) kits (Applied Biosystems; Thermo Fisher Scientific) were used to detect porcine circovirus type 2 (PCV2), PCV3, pseudorabies virus (PRV), PRRSV, classical swine fever virus (CSFV), and porcine parvovirus (PPV) as previously described [22–25], following the manufacturer’s protocols.

Lung tissue homogenates in PBS were centrifuged at 12,000 × *g* for 10 min at 4°C. The supernatants were filtered through a 0.22‐μm filter (Millipore Sigma) and inoculated onto primary PAMs for virus isolation. Cells were monitored daily for cytopathic effects (CPEs), and PRRSV was confirmed by an indirect immunofluorescence assay (IFA).

The isolated virus was purified by the plaque assay in MARC‐145 cells as previously described [7]. Briefly, MARC‐145 cells in 6‐well plates (80% confluence) were inoculated with 200 μL of 10‐fold serially diluted second‐passage virus supernatant for 1 h at 37°C. After two washes with sterile PBS (pH 7.2), cells were overlaid with 2 mL of Dulbecco’s minimal Eagle’s medium (DMEM) containing 2% FBS and 1% low‐melting‐point agarose. Following solidification, plates were inverted and incubated at 37°C. Almost 5 days postinfection, individual plaques were picked under a microscope and transferred to fresh MARC‐145 cell monolayers in 6‐well plates and then cultured in DMEM with 2% FBS for 3–5 days. Culture supernatants were collected after centrifugation at 3000 × g for 20 min. After two rounds of plaque purification and one additional propagation passage, the purified virus was designated PRRSV AHFY. Viral stocks were stored at −80°C, and viral particles were observed by transmission electron microscopy (TEM) (Chengdu Lilai Biotechnology Co., Ltd.).

### 2.3. Indirect IFA and Next‐Generation Sequencing (NGS)

An indirect IFA was performed to confirm PRRSV isolation as previously described [21] with minor modifications. Briefly, MARC‐145 cells inoculated with the purified PRRSV strain were washed twice with PBS at 24 h postinoculation (hpi), fixed in cold ethanol at 4°C for 30 min, and blocked with 1% BSA for 1 h at room temperature (RT). Cells were then incubated with the 6D10 mAb against the PRRSV N protein (primary antibody) for 1 h at 37°C, followed by FITC‐labeled goat anti‐mouse secondary antibody (Jackson ImmunoResearch, West Grove, PA, USA). Nuclei were counterstained with 4′, 6‐diamidino‐2‐phenylindole (DAPI), and fluorescence was observed under a fluorescence microscope (Leica, Wetzlar, Germany).

Total viral RNA was extracted from PRRSV‐infected cell suspensions using the E.Z.N.A. Viral RNA Kit (Omega, USA) and subjected to NGS on an Illumina platform [26]. Briefly, RNA samples were fragmented and randomly reverse‐transcribed into cDNA. Sequencing adapters were ligated to both ends of the cDNA fragments, and the libraries were amplified by bridge‐PCR. Paired‐end (140 bp) sequencing was performed on an Illumina NovaSeq6000 platform. De novo assembly was carried out using SPAdes v3.14.1, and assembled contigs were filtered to retain sequences ≥100 bp. The best BLAST hits against the NT database were obtained, and the complete PRRSV genome sequence was annotated based on the SwissProt database. Multiple sequence alignments were performed using MegAlign (version 7.1) in the Lasergene software package (DNASTAR, Madison, WI, USA).

### 2.4. Genetic Recombination Analysis

Potential recombination events between the isolated PRRSV strain and representative reference strains (VR‐2332, QYYZ, JXA1, CH‐1a, NADC30, NADC34, CH‐1R, JXA1‐R, and TJM‐F92) were analyzed using RDP4 software with seven detection methods: RDP, GENECONV, BootScan, MaxChi, Chimaera, SiScan, and 3Seq. A recombination event was considered valid only if supported by at least five of the seven methods, each with a significance threshold of *p*  < 1 × 10^−6^. Recombination breakpoints were further validated by sliding window analysis (200 bp window, 20 bp step size) using SimPlot 3.5.1 with default parameters.

### 2.5. Virus Culture, Serial Passage Attenuation, and NGS Across Successive Passages

The PRRSV isolate AHFY was serially passaged 140 times in MARC‐145 cells using DMEM (Life Technologies Corporation, Grand Island, NY, USA) supplemented with 3% FBS (GIBCO, Brazil) at 37°C in a humidified 5% CO_2_ incubator. Cells were monitored daily for CPEs. When ~80% CPE was observed, cells were harvested, freeze‐thawed three times, and centrifuged at 10,000 × *g* for 10 min at 4°C. The supernatants were collected to inoculate fresh MARC‐145 cells and stored at −80°C. This process was repeated for up to 140 passages (F1–F140).

Virus purification by plaque assay in MARC‐145 cells (as described above) and viral titration were performed every 10 passages. For titration, viral stocks were serially 10‐fold diluted in DMEM and added to MARC‐145 cells in 96‐well plates (100 μL/well). After incubation at 37°C for 7 days, the 50% tissue culture infectious dose (TCID_50_) was calculated using the Reed–Muench method [27]. The 140th‐passage virus was designated AHFY‐F140 and used for subsequent evaluations.

Total viral RNA was also extracted from every 10th passage for NGS the Illumina technology, as described earlier.

### 2.6. Comparison of the Pathogenicity of the 1st and 140th Passages of PRRSV AHFY in Piglets

Fifteen 4‐week‐old PRRSV‐free New American Line piglets (Shandong Xincheng Animal Husbandry Technical Service Co., Ltd., China) were confirmed negative for PRRSV RNA and antibodies. Written informed consent was obtained from the farm owner. Piglets were randomly divided into three groups (*n* = 5 per group). Group 1 was intramuscularly inoculated with 10^5.0^ TCID_50_ of the AHFY strain at passage 1 (AHFY‐F1). Group 2 received the same dose of the 140th‐passage virus (AHFY‐F140). Group 3 served as the unvaccinated control. All animal experiments were conducted in a biosafety level 2 (BSL‐2) facility (Shandong Sinder Technology Co., Ltd.), with each group housed separately.

Clinical signs (coughing, dyspnea, anorexia, diarrhea, lameness, shivering, and fever) were scored daily using a 0–6 system (0 = normal; 6 = severe dyspnea with abdominal breathing) [28]. Rectal temperature and body weight were recorded throughout the experiment. Serum samples were collected at 0, 1, 3, 5, 7, 9, 11, 14, 18, and 21 days postinoculation (dpi) for viremia assessment by reverse transcription‐quantitative PCR (RT‐qPCR) [29].

For RT‐qPCR, total RNA was extracted from serum using the TRIzol reagent (Invitrogen; Thermo Fisher Scientific) and reverse‐transcribed into cDNA using the PrimeScript RT Reagent Kit (TaKaRa Bio) following the manufacturer’s protocol. qPCR was performed on a StepOne Plus Real‐Time PCR System (Applied Biosystems; Thermo Fisher Scientific) using FastStar Universal SYBR Green Master Mix (Roche) in a 10 μL reaction. The cycling conditions were 95°C for 10 min, followed by 40 cycles of 95°C for 15 s and 60°C for 1 min. Viral RNA in serum was quantified using the following primers and probes: forward 5′—AAAAATTGCATGTCCTGGCG—3′, reverse 5′—CAACATCAACTTTACCCCCT—3′, and probe 5′—[FAM]AACTCTATCGTTGGCGGTCGCCTGT[BHQ‐1]—3′.

PRRSV‐specific antibodies in the serum were detected using a commercial ELISA kit (IDEXX Laboratories, Westbrook, ME, USA) according to the manufacturer’s instructions.

All piglets were euthanized at 21 dpi. Lung tissues were collected for viral load determination by RT‐qPCR, as described above. For histopathological examination, lung samples were fixed in 4% paraformaldehyde, embedded in paraffin, sectioned, stained with hematoxylin and eosin (H&E), and examined under a light microscope.

### 2.7. Evaluation of the Immunogenicity and Protective Efficacy of the PRRSV AHFY‐F140 Strain

Twenty‐five 4‐week‐old PRRSV‐free New American Line piglets (Shandong Xincheng Animal Husbandry Technical Service Co., Ltd., China) were randomly divided into five groups (*n* = 5 per group). All piglets tested negative for PRRSV RNA and antibodies. Piglets in Groups 1 and 2 were intramuscularly inoculated with 10^5^·^0^ TCID_50_ of AHFY‐F140. Groups 3, 4, and 5 were inoculated with DMEM and served as control groups. Animals in different groups were housed in separate rooms.

At 21 days postvaccination (dpv), piglets in Groups 1 and 3 were challenged intramuscularly (neck) with 2 mL of the AHFY‐F1 strain (10^5^·^0^ TCID_50_ per pig). Piglets in Groups 2 and 4 received the same dose of the JXA1 (HP‐PRRSV) strain. Group 5 (mock) was inoculated with an equal volume of PBS.

The rectal temperature was recorded daily. Body weight and serum samples were collected at 1, 3, 5, 7, 9, 11, 13, 15, 17, 19, and 21 days postchallenge (dpc). All piglets were euthanized with sodium pentobarbital at 21 dpc, and lung tissues were collected as described earlier.

### 2.8. Statistical Analysis

Statistical analyses were performed using GraphPad Prism 6.01 (GraphPad Software, CA, USA). Data are presented as the mean ± standard deviation (SD). Differences were considered statistically significant at a *p* < 0.05. Significance levels are indicated as follows:  ^∗∗^ *p*  < 0.01,  ^∗∗∗^ *p*  < 0.001,  ^∗∗∗∗^ *p*  < 0.0001.

## 3. Results

### 3.1. Virus Isolation, Identification, and Purification

**Figure 1:**
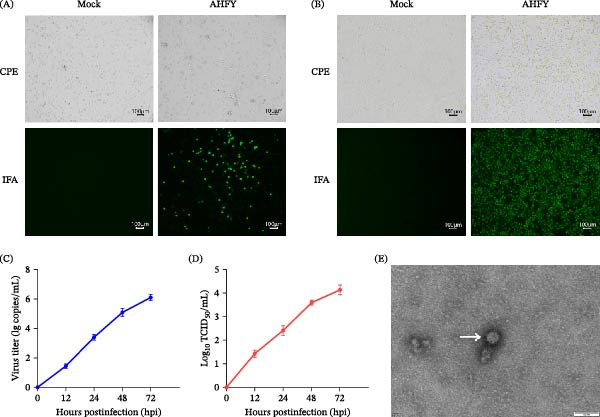
Isolation and characterization of the PRRSV AHFY strain from a piglet lung sample. (A) Primary alveolar macrophages (PAMs) were inoculated with lung homogenate. Cytopathic effect (CPE) was observed at 72 hpi. Expression of PRRSV N protein in PAMs was confirmed by immunofluorescence assay (IFA) using an anti‐N monoclonal antibody. (B) MARC‐145 cells showing CPE and PRRSV N protein detection by IFA after AHFY infection. (C) Virus titer (lg copies/mL) and (D) log_10_TCID_50_/mL of AHFY in MARC‐145 cells at 12, 24, 48, and 72 hpi. (E) Transmission electron micrograph of purified AHFY virions. Scale bar: 100 nm.

RT‐qPCR‐based nucleic acid screening of lung tissue samples detected only PRRSV RNA; tests for PCV2, PRV, CSFV, and PPV were all negative. Supernatants from PRRSV RNA‐positive tissue homogenates were processed aseptically and inoculated onto PAMs for virus isolation. Subsequent propagation in MARC‐145 cells allowed for further biological characterization of the isolate. Inoculated PAMs developed CPEs within 48 hpi, characterized by cell shrinkage, membrane blebbing, and partial monolayer detachment (Figure 1A). An indirect IFA using PRRSV‐N‐specific monoclonal antibodies confirmed viral replication, as distinct cytoplasmic fluorescence was observed in inoculated PAMs, while no fluorescence was detected in negative control cells (Figure 1A). These results confirmed the successful isolation of a PRRSV strain from clinical specimens, which was designated AHFY.

When MARC‐145 cells were inoculated with the PRRSV AHFY strain, CPEs were observed at 48 hpi, featuring cell aggregation, cytoplasmic shrinkage, and monolayer detachment. Uninfected control cells maintained a normal morphology with no CPEs. IFA of inoculated MARC‐145 cells showed bright cytoplasmic fluorescence when probed with PRRSV‐N‐specific antibodies, whereas control cells exhibited no specific signal (Figure 1B). Viral replication kinetics analysis revealed a peak viral RNA titer of 10^6^·^3^ copies/mL (Figure 1C) and a maximum infectious titer of 10^4^·^3^ TCID_50_/mL at 72 hpi (Figure 1D). TEM further confirmed the presence of enveloped, spherical PRRSV virions with a diameter of ~ 50–70 nm in purified supernatants (Figure 1E).

NGS and assembly revealed the complete genome of PRRSV strain AHFY to be 15,022 nts in length. This sequence has been deposited in GenBank with the Accession Number PQ285382. Comparative genomic analysis showed that AHFY shared nt identity >80% with representative PRRSV‐2 strains, including VR‐2332 (lineage 5), JXA1 (lineage 8), NADC30 (lineage 1.8), and QYYZ (lineage 3). In contrast, it exhibited only 60.3% nt identity to the PRRSV‐1 prototype strain, Lelystad virus (Table 1).

**Table 1:** Genomic identity of the whole genome between the AHFY strain and representative strains (%).

Strains	AHFY	VR2332 (lineage 5)	JXA1 (lineage 8)	NADC30 (lineage 1.8)	QYYZ (lineage 3)	LV (PRRSV‐1)
AHFY	^∗^	84.6	84.8	89.9	83.2	60.3
VR2332 (lineage 5)	84.6	^∗^	89.6	86.6	86.3	60.0
JXA1 (lineage 8)	84.8	89.6	^∗^	84.7	87.8	59.9
NADC30 (lineage 1.8)	89.9	86.6	84.7	^∗^	82.9	60.7
QYYZ (lineage 3)	83.2	86.3	87.8	82.9	^∗^	59.5
LV (PRRSV‐1)	60.3	60.0	59.9	60.7	59.5	^∗^

*Note*:  ^∗^ Compared with the same strain; blank cells indicate no data available.

Phylogenetic analysis was performed using the neighbor‐joining method (1000 bootstrap replicates) based on 36 full‐length PRRSV genomes, which clearly separated the two distinct PRRSV genotypes (PRRSV‐1 and PRRSV‐2). Within the PRRSV‐2 clade, strain AHFY clustered closely with the NADC30‐like strain JL580 (lineage 1.8), showing clear genetic divergence from lineage 3 (QYYZ) and lineage 5 (VR‐2332) (Figure 2A). Phylogenetic analysis confirmed that AHFY belongs to lineage 1.8 (NADC30‐like).

Figure 2Phylogenetic analysis of different PRRSV isolates based on complete genome sequences and amino acid alignment analysis of NSP2. (A) A neighbor‐joining tree was constructed using MEGAX. The AHFY strain (red marker, 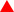) clusters within the NADC30‐like lineage (lineage 1.8). Bootstrap values (1000 replicates) are shown for key nodes. (B) Amino acid alignment of NSP2 from NADC30‐like strains. AHFY exhibits a characteristic 131‐amino acid deletion (positions 323–433, 483, and 504–522) being consistent with lineage 1.8. (C) Whole‐genome analysis identified PRRSV‐AHFY as a quadruple‐parent recombinant, derived from NADC30 (lineage 1) with genomic insertions from CH‐1R, TJM‐F92, and QYYZ.

**Figure:**
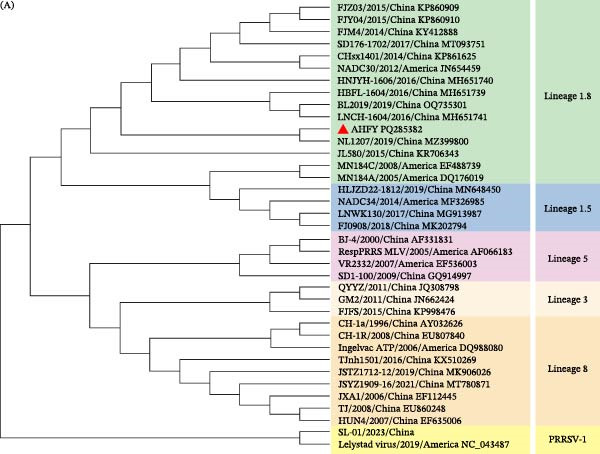

**Figure:**
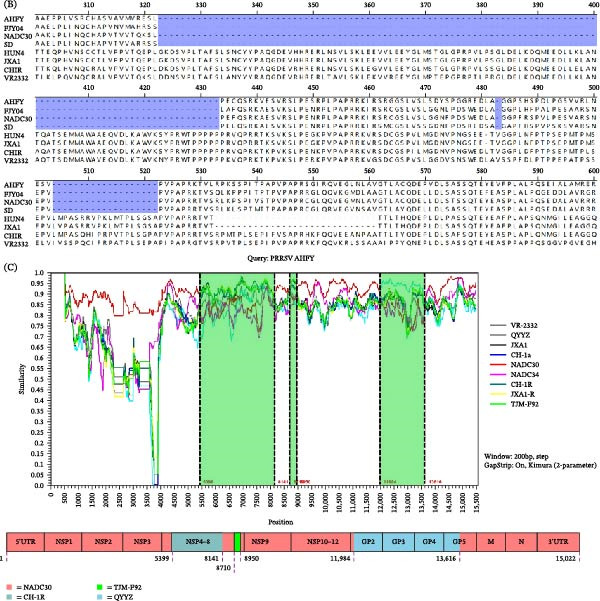

Further analysis of the NSP2 gene identified a 2241 bp coding sequence, encoding 747 aas, which contained characteristic deletions. Sequence alignment with VR‐2332 (lineage 5) revealed discontinuous deletions totaling 131 aas: a 111‐aa deletion at positions 323–433 and a 19‐aa deletion at positions 499–517 (Figure 2B). These structural features are consistent with the established molecular signature of NADC30‐like strains, further supporting their lineage classification (Figure 2B).

### 3.2. Recombination Patterns Define PRRSV‐AHFY as a Quadruple‐Parent Recombinant

Whole‐genome analysis identified strain AHFY as a quadruple‐parent recombinant, derived from NADC30 (lineage 1) with genomic insertions from CH‐1R (positions 5399‐8141 bp), TJM‐F92 (8710‐8950 bp), and QYYZ (11,984‐13,616 bp) (Figure 2C). These recombination breakpoints were validated by RDP4 software using four or more detection algorithms, each meeting a significance threshold of *p*  < 1 × 10^–6^. SimPlot visualization further confirmed the crossover boundaries (Table 2). Phylogenetic analysis of individual genome regions revealed distinct lineage affiliations. The 5′ UTR, NSP1‐3, NSP9‐12, GP5, M, N, and 3′ UTR regions were predominantly derived from NADC30‐like (lineage 1) sequences. In contrast, the recombinant fragments within the NSP4‐8 and GP2‐4 regions exhibited high sequence identity to strains CH‐1R (lineage 8), TJM‐F92 (lineage 8), and QYYZ‐like (lineage ly. This chimeric genomic architecture indicated that strain AHFY was a complex recombinant strain that integrated genetic elements from four epidemiologically and genetically distinct PRRSV lineages.

**Table 2:** Possible recombination events between AHFY and other representative strains in RDP4.

GRE	Major parental	Minor parental	Site/bp	Region	Seven recombination detection methods and *p*‐values						
					RDP	GENECONV	BootScan	MaxChi	Chimaera	SiScan	3Seq
1	NADC30	CH‐1R	5399–8141	NSP4‐8	3.792 × 10^−44^	—	1.241 × 10^−67^	6.255 × 10^−25^	3.739 × 10^−28^	4.18 × 10^−20^	1.967 × 10^−58^
2	NADC30	TJM‐F92	8710–8950	NSP4‐8	7.376 × 10^−11^	7.775 × 10^−08^	6.654 × 10^−10^	1.275 × 10^−02^	2.528 × 10^−03^	—	—
3	NADC30	QYYZ	11,984–13,616	GP2‐5	4.497 × 10^−70^	9.767 × 10^−38^	2.575 × 10^−68^	2.831 × 10^−25^	9.012 × 10^−28^	8.647 × 10^−28^	3.04 × 10^−29^

*Note:* The recombination position is based on the AHFY strain sequence.

### 3.3. Mutational Dynamics and Replication Kinetics of Serially Passaged AHFY in MARC‐145 Cells

**Figure 3:**
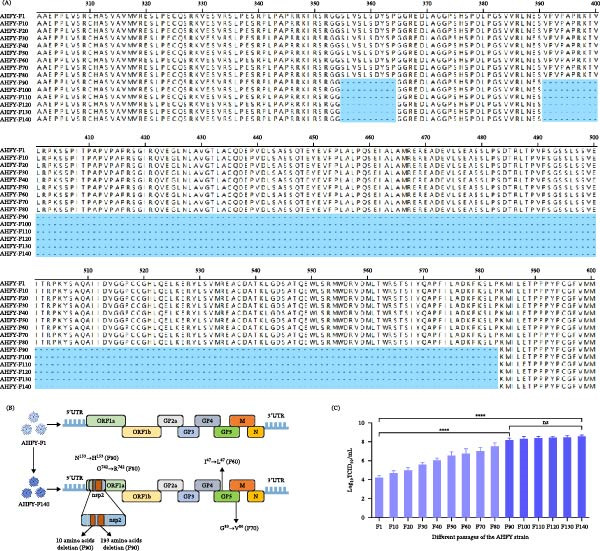
The dynamic changes of NSP2 gene deletions, amino acid mutations, and viral replication capacity during the serial passage of the AHFY strain from F1 to F140. (A) Serial passage (F1–F140) analysis of NSP2 deletions, stable dual deletions (10 aa: 355–364; 193 aa: 391–583) emerged by passage 90 (F90). (B) Amino acid mutations in AHFY‐F140 compared to F1. (C) The viral titer of AHFY strain in MARC‐145 cells was determined to progressively increase with passage number by assessing viral replication kinetics through TCID_50_ assay. ^∗∗∗∗^ *p* < 0.0001.

Consistent with previous reports on PRRSV genomic plasticity [30, 31], we identified mutational hotspots in the NSP2 and GP5 proteins across 140 serial passages (F1–F140) of AHFY in MARC‐145 cells. aa sequencing revealed two stable mutations in NSP2: N153H (asparagine to histidine) emerged at passage 90 and G742R (glycine to arginine) was fixed by passage 80 (Table 3). Notably, two large continuous deletions in NSP2—10 aas (positions 355–364) and 193 aas (positions 391–583)—became apparent from passage 90 onward (Figure 3A). In GP5, sequential substitutions—I47L (isoleucine to leucine) and G80V (glycine to valine)—were detected at passages 60 and 70, respectively (Figure 3B and Table 3).

**Table 3:** Amino acid mutation analysis of NSP2 gene and GP5 gene of different generations of AHFY virus.

Generation	NSP2				GP5	
	153	355–364	391–583	742	47	80
F1	N	10 amino acids	193 amino acids	G	I	G
F10	N	10 amino acids	193 amino acids	G	I	G
F20	N	10 amino acids	193 amino acids	G	I	G
F30	N	10 amino acids	193 amino acids	G	I	G
F40	N	10 amino acids	193 amino acids	G	I	G
F50	N	10 amino acids	193 amino acids	G	I	G
F60	N	10 amino acids	193 amino acids	G	L	G
F70	N	10 amino acids	193 amino acids	G	L	V
F80	N	10 amino acids	193 amino acids	R	L	V
F90	H	Deletion	Deletion	R	L	V
F100	H	Deletion	Deletion	R	L	V
F110	H	Deletion	Deletion	R	L	V
F120	H	Deletion	Deletion	R	L	V
F130	H	Deletion	Deletion	R	L	V
F140	H	Deletion	Deletion	R	L	V

Viral replication kinetics were evaluated via the TCID_50_ assay, which showed progressive adaptation of the virus to MARC‐145 cells. The parental strain AHFY‐F1 had a low initial titer (10^4^·^3^ TCID_50_/mL), while the peak replication efficiency (10^8^·^7^ TCID_50_/mL) was reached by passage 90. Viral titers remained stable in subsequent passages (F90–F140), indicating genomic stabilization (Figure 3C).

### 3.4. Comparison of the Pathogenicity of PRRSV AHFY‐F1 and AHFY‐F140 in Piglets

**Figure 4:**
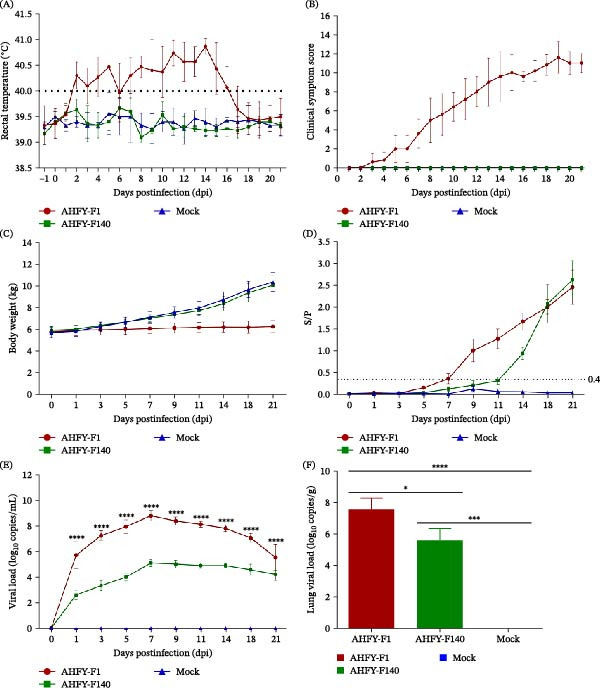
Clinical and virological outcomes of piglets inoculated with PRRSV virulent strain AHFY‐F1 or attenuated strain AHFY‐F140. (A) Rectal temperature measured continuously after challenge, (B) clinical symptom scores (0–6 scale) of piglets in each group postchallenge, and (C) body weight changes in the challenged groups (AHFY‐F1, AHFY‐F140) and mock group. (D) Serum anti‐PRRSV antibody levels, expressed as S/P ratio. (E) Viremia in piglets quantified by RT‐qPCR, with units of log_10_ copies/mL; data are presented as mean ± SEM (*n* = 5 per group). (F) Viral copy numbers in lung tissues of each piglet detected by RT‐qPCR, with units of log_10_ copies/g.  ^∗^ *p*  < 0.05,  ^∗∗^ *p*  < 0.01,  ^∗∗∗^ *p*  < 0.001, and  ^∗∗∗∗^ *p* < 0.0001.

Piglets inoculated with the virulent AHFY‐F1 strain developed acute clinical signs, including sustained pyrexia (≥ 40.0°C for 7–14 days; Figure 4A), lethargy, tachypnea, abdominal breathing, periorbital edema, and cyanotic cutaneous lesions. In contrast, AHFY‐F140‐inoculated piglets remained clinically asymptomatic with normal body temperatures (< 40.0°C; Figure 4A), consistent with the mock group. Quantitative clinical scoring showed significantly higher clinical severity in AHFY‐F1‐inoculated animals compared to the AHFY‐F140 and mock groups (Figure 4B).

Notably, AHFY‐F140‐inoculated piglets had weight gain trajectories similar to those of mock‐inoculated control piglets, whereas AHFY‐F1 inoculation induced significant growth retardation (Figure 4C). Serological analysis showed distinct antibody kinetics between the groups. In the AHFY‐F1 group, one out of five piglets seroconverted by 7 dpi, with full seropositivity achieved by 9 dpi. For the AHFY‐F140 group, initial seroconversion occurred at 11 dpi (1/5 animals), reaching 100% seropositivity by 14 dpi (Figure 4D). Control piglets remained seronegative throughout the 21‐day observation period.

Viral load dynamics in the serum were quantified using RT‐qPCR [29]. Peak viremia was observed at 7 dpi in the AHFY‐F1‐inoculated group and at 9 dpi in the AHFY‐F140‐inoculated group. However, AHFY‐F140‐inoculated piglets exhibited substantially lower peak viral titers (10^5^·^9^ RNA copies/mL) compared to their AHFY‐F1‐inoculated counterparts (10^9^·^0^ RNA copies/mL), with progressive viral clearance thereafter (Figure 4E). Furthermore, lung viral loads were quantified at 21 dpi to further evaluate the pathogenicity across all groups. Viral RNA levels in the lungs of AHFY‐F1‐inoculated piglets (10^7^·^6^ copies/g) were significantly higher than those in AHFY‐F140‐inoculated piglets (10^5^·^6^ copies/g), whereas no viral RNA was detected in the Mock group; all statistical comparisons showed significant differences (*p*  < 0.05; Figure 4F).

**Figure 5:**
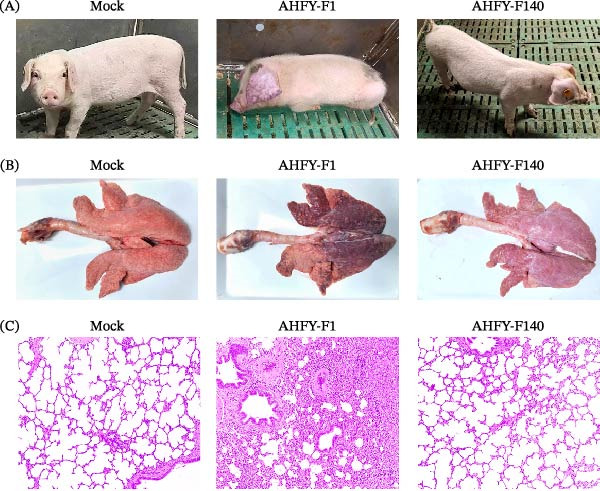
Pathological manifestations in challenged piglets. (A) Clinical signs (dyspnea and lethargy). (B) Gross lung lesions: interstitial pneumonia and congestion. (C) Histopathology (H&E staining): bronchiolar inflammation and alveolar septal thickening. Scale bars: 200 μm (lung tissue).

All five piglets in the AHFY‐F1‐inoculated group showed clinical and pathological signs of PRRSV infection, including persistent hyperthermia (≥40.0°C), lethargy, recumbency, cyanotic ear margins, and anorexia (Figure 5A). Gross pathological examination revealed severe pulmonary lesions, including diffuse interstitial pneumonia, marked lung edema, multifocal consolidation (mid‐to‐caudal lobes), and a firm, rubber‐like lung parenchymal consistency (Figure 5B). Histopathological analysis confirmed interstitial pneumonia, characterized by alveolar septal thickening, mononuclear inflammatory cell infiltration (predominantly lymphocytes and macrophages), type II pneumocyte hyperplasia, and intra‐alveolar proteinaceous exudates (Figure 5C).

In contrast, no clinical signs or gross/histopathological abnormalities were observed in AHFY‐F140‐inoculated or mock groups (Figures 5A,B,C). These results confirmed that the AHFY‐F140 strain was fully attenuated, verifying its safety profile in susceptible piglets.

### 3.5. Clinical Evaluation After Immunization and Challenge

**Figure 6:**
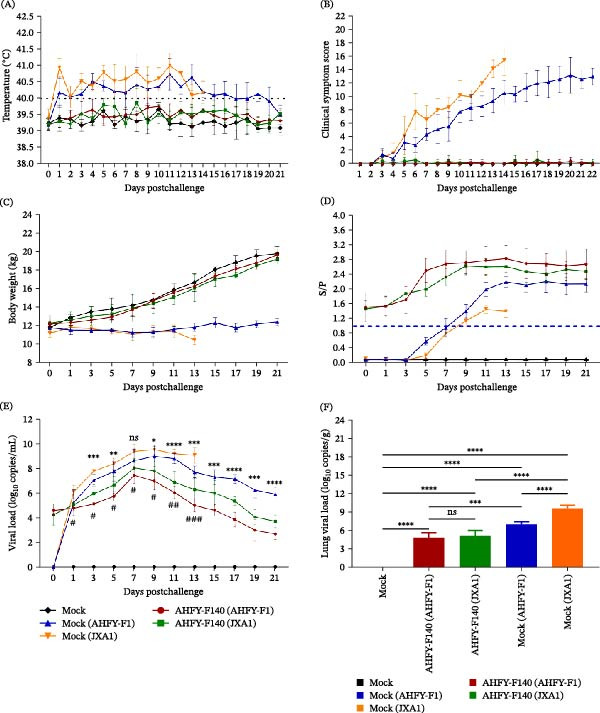
Protective efficacy comparison of PRRSV AHFY‐F1 and attenuated AHFY‐F140 in a vaccine immunization‐challenge trial. (A) Comparison of body temperature changes induced by AHFY‐F1 and AHFY‐F140. (B) Clinical scores of pigs challenged with AHFY‐F1 and AHFY‐F140. (C) Body weight changes of pigs after inoculation with AHFY‐F1 and AHFY‐F140. (D) Serum antibody responses of pigs infected with AHFY‐F1 and AHFY‐F140. (E) Blood viral loads in AHFY‐F1‐ and AHFY‐F140‐challenged pigs. (F) Lung viral loads in AHFY‐F1‐ and AHFY‐F140‐inoculated pigs. ^∗^ indicates significant differences between Mock (AHFY‐F1) and AHFY‐F140 (AHFY‐F1),  ^∗^ *p* < 0.05,  ^∗∗^ *p* < 0.01,  ^∗∗∗^ *p* < 0.001,  ^∗∗∗∗^ *p* < 0.0001. ^#^ indicates significant differences between Mock (JXA1) and AHFY‐F140 (JXA1), ^#^ *p* < 0.05, ^##^ *p* < 0.01, ^###^ *p* < 0.001, ^####^ *p* < 0.0001.

During the experiment, no PRRS‐related clinical signs—including changes in behavior, body temperature, or body weight—were observed in piglets from the AHFY‐F140 (AHFY‐F1) group, AHFY‐F140 (JXA1) group, and the Mock group (data not shown). In contrast, piglets in the Mock (AHFY‐F1) and Mock (JXA1) groups developed various disease manifestations, including persistent fever (≥ 40.5°C) from 7–14 dpc (Figure 6A), as well as anorexia, skin cyanosis, chemosis, coughing, shivering, or lameness (Figure 6B). Notably, all piglets in the mock (JXA1) group died by 14 dpc.

In particular, with regard to body weight, piglets in the AHFY‐F140 (AHFY‐F1) group, AHFY‐F140 (JXA1) group, and mock group gained weight significantly faster (*p*  < 0.0001) than those in the mock (AHFY‐F1) and mock (JXA1) groups (Figure 6C).

### 3.6. Antibody Responses in Challenged Piglets

Detection of PRRSV‐specific antibodies using a commercial ELISA kit revealed that antibodies were already present in the sera of piglets from the AHFY‐F140 (AHFY‐F1) group and AHFY‐F140 (JXA1) group at 0 dpc. In contrast, all piglets in the mock (AHFY‐F1) and mock (JXA1) groups seroconverted by 9 dpc (Figure 6D). Piglets in the mock group remained seronegative for PRRSV antibodies throughout the experiment (Figure 6D).

To evaluate viremia and the duration of PRRSV infection, serum samples collected at 0, 1, 3, 5, 7, 9, 11, 13, 15, 17, 19, and 21 dpc were analyzed using RT‐qPCR. In both the mock (AHFY‐F1) and mock (JXA1) groups, serum PRRSV RNA was first detected at 1 dpc, with peak viral titers observed at 9 dpc and 7 dpc, respectively.

### 3.7. Virus Detection in Challenged Piglets

Peak viremia was reached at 7 dpc in both the AHFY‐F140 (AHFY‐F1) and AHFY‐F140 (JXA1) groups. After reaching the peak, serum viral titers decreased slightly. Piglets in these two groups exhibited substantially lower peak viral titers compared to those in the mock (AHFY‐F1) and mock (JXA1) groups, with progressive viral clearance thereafter (Figure 6E).

Furthermore, lung viral loads were quantified at 21 dpc to further evaluate the pathogenicity across all groups. Viral RNA levels in lung tissues were significantly elevated in both the mock (JXA1) and mock (AHFY‐F1) groups, measuring 10^9^·^6^ copies/g and 10^7^·^0^ copies/g, respectively, with a highly significant difference between these two groups (*p*  < 0.0001). Moreover, compared with the mock group (in which no viral RNA was detected), both groups showed extremely significant statistical differences (*p*  < 0.0001). Compared with the mock (AHFY‐F1) group, viral loads in lung tissues of piglets in the AHFY‐F140 (AHFY‐F1) group (10^4^·^8^ copies/g) were significantly lower (*p*  < 0.001). Similarly, compared with the mock (JXA1) group, viral loads in lung tissues of piglets in the AHFY‐F140 (JXA1) group (10^5^·^1^ copies/g) were also significantly lower (*p*  < 0.001). No significant difference in lung viral loads was observed between the AHFY‐F140 (AHFY‐F1) and AHFY‐F140 (JXA1) groups; however, both groups showed extremely significant differences compared to the mock group (*p*  < 0.001; Figure 6F).

### 3.8. Pathological and Histopathological Examination

**Figure 7:**
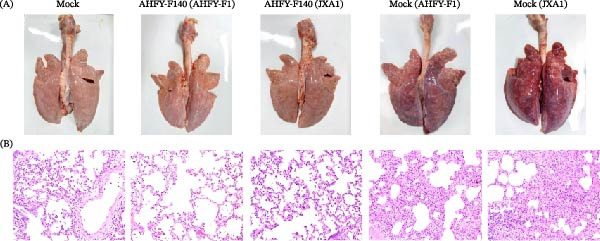
Observation of lung lesions in the vaccine immunization‐challenge trial to evaluate the protective efficacy of PRRSV attenuated strain AHFY‐F140 against challenges with virulent strains AHFY‐F1 and JXA1. (A) Gross pathological examination of lung tissues. (B) Histopathological observation of lung lesions by HE staining. Scale bars: 200 μm (lung tissue).

Compared with piglets in the AHFY‐F140 (AHFY‐F1) and AHFY‐F140 (JXA1) groups, those in the mock (AHFY‐F1), mock (JXA1), and mock groups exhibited typical PRRS‐associated gross pulmonary lesions, including consolidation, firm, dense parenchymal tissue, and hemorrhage in the lungs (Figure 7A,B). Histological analysis revealed extensive inflammatory cell infiltration and significant thickening of the alveolar septa in the lungs of piglets in the mock (AHFY‐F1) and mock (JXA1) groups (Figure 7C,D). In contrast, no microscopic lesions were observed in the AHFY‐F140 (AHFY‐F1) and AHFY‐F140 (JXA1) groups (Figure 7C,D). These data indicate that PRRSV strain AHFY‐F140 can reduce the incidence of gross and microscopic pulmonary lesions in vaccinated piglets following challenge with the NADC30‐like AHFY‐F1 strain and the JXA1 (HP‐PRRSV) strain.

## 4. Discussion

Since 2013, the emergence of NADC30‐like PRRSV strains (lineage 1) in China, characterized by a consistent 131‐aa deletion in the NSP2 protein, has caused substantial economic losses in the swine industry [32]. These strains have become endemic and now account for a large proportion of circulating PRRSV variants across China [33–35]. Comparative genomic analyses have revealed considerable heterogeneity in virulence among NADC30‐like isolates. For instance, strains such as 14LY01‐FJ and JL580 are highly virulent, whereas others, including FJ1402 and SD53−1603 show moderate virulence [36–39]. In the present study, the AHFY strain exhibited a moderate virulence phenotype similar to FJ1402 and SD53‐1603 but induced a more prolonged febrile response (15 days vs. 9–12 days for other strains). This phenotypic diversity highlights the urgent need to develop vaccines specifically targeted against these emerging variants [14].

Current PRRSV control strategies rely heavily on MLVs derived from HP‐PRRSV lineage 8 strains (e.g., JXA1‐R and TJM‐F92), which were attenuated via long‐term serial passage in MARC‐145 cells [40]. Notably, a stable 120‐aa deletion (aa 628–747) in NSP2 appeared in TJM‐F92 by passage 15. However, the limited genomic homology between HP‐PRRSV and prevalent NADC30‐like strains severely compromises the cross‐protective efficacy, leading to frequent vaccine failures in the field [14]. To address this gap, we generated an attenuated vaccine candidate, AHFY‐F140, by serially passaging the NADC30‐like isolate AHFY for 140 generations in MARC‐145 cells. The AHFY genome became stable after passage 90, and the viral titer increased by over 10^4^‐fold, indicating complete cellular adaptation. Accompanying this attenuation process, AHFY‐F140 stably acquired two discontinuous large deletions in the NSP2 hypervariable region: a 10‐aa deletion at residues 355–364 and a 193‐aa deletion at residues 391–583, as well as two aa substitutions in GP5 (I47L and G80V). The parental AHFY strain already carries the lineage‐1.8‐characteristic 131‐aa deletion (residues 323–433, 485, 499–517), and the additional deletions, together with the GP5 mutations, emerged during passaging and likely contribute to attenuation.

Large fragment deletions in the NSP2 region are molecular features that may occur during the attenuation process of PRRSV strains adapting to the cell culture. Song et al. [18] demonstrated that deletion of the NSP2 hypervariable region (aa 323–521) in the HP‐PRRSV JXwn06 strain abolished viral infectivity in primary PAMs, suggesting that such deletions are key events in attenuation. Kong et al. [19] further showed that lineage‐specific NSP2 variations directly modulate virulence and persistence, with the lineage‐1 131‐aa deletion and the HP‐PRRSV 30‐aa deletion serving as stable markers. Bai et al. [16, 17] reported that NSP2 in commercial attenuated vaccines (e.g., CH‐1R) acquires multiple deletions and mutations that cause an uncoating defect in PAMs and that full‐length NSP2 acts as a bridging molecule in viral assembly, a function disrupted by NSP2 truncation. Given the established role of NSP2 in PRRSV virulence, the acquisition of these large deletions during attenuation strongly suggests their contribution to the reduced virulence of AHFY‐F140 [26]. Therefore, the novel dual deletion in AHFY‐F140 NSP2 not only provides a unique molecular marker for differentiating this candidate from wild‐type viruses—a critical feature for epidemiological surveillance—but also likely contributes to attenuation by impairing viral replication and assembly in primary target cells.

Regarding GP5, the G80V substitution disrupts the conserved immunoreceptor tyrosine‐based inhibitory motif (ITIM, ^77^VSYGAL^82^), which is known to suppress host immune responses. This mutation may relieve inhibitory signaling and enhance the host immune response. The I47L substitution serves as a lineage 1‐specific marker, although its functional role requires further investigation. Notably, the aa changes in GP5 are consistent with mutations found in the commercial vaccine strain RespPRRS MLV (derived from VR2332), further supporting a role in attenuation.

Several other NADC30‐like vaccine candidates have been explored. Guo et al. [41] obtained the attenuated strain GXHX20211106‐P100 after 100 passages, achieving a titer of 10^7^·^36^ TCID_50_/mL; immunization reduced homologous viremia and lung lesions but did not significantly inhibit mucosal shedding. Zhou et al. [42] constructed a GP5‐modified LNP mRNA vaccine that induced IFN‐γ^+^ T‐cell responses and reduced viral load but did not elicit neutralizing antibodies. Li et al. [43] reported that pigs immunized with commercial PRRSV vaccines became infected with two NADC30‐like viruses, demonstrating that current commercial vaccines cannot prevent NADC30‐like infection, one isolate recombined with VR‐2332 and CH‐1a, but no attenuated vaccine was developed from these viruses. For the SD strain, Zhang et al. [44] performed 125 passages to generate the attenuated SD‐R, which conferred complete protection against homologous SD and heterologous HLJWK108‐1711 challenges, and Li et al. [45] subsequently showed that SD‐R reduced thymic atrophy and pneumonia caused by an HP‐like strain. In comparison, AHFY‐F140 presents several distinct features: (i) it carries a unique discontinuous dual deletion in NSP2 (10‐aa + 193‐aa) not reported in other attenuated strains; (ii) its viral titer (10^8^·^7^ TCID_50_/mL) is higher than that of GXHX20211106‐P100; (iii) unlike the mRNA vaccine, AHFY‐F140 elicited protection against the heterologous HP‐PRRSV JXA1 strain (lineage 8); (iv) compared with SD‐R, AHFY‐F140 was derived through more passages (140 vs. 125), possesses a novel deletion signature, and additionally provides cross‐protection against the highly virulent JXA1 strain. The failure of commercial vaccines against NADC30‐like viruses further underscores the urgency for lineage‐matched MLVs such as AHFY‐F140 [43]. In addition, we will further construct an infectious clone of AHFY‐F140 to clarify the effects of these specific deletion or mutation sites on viral virulence and replication kinetics.

Nevertheless, recombination remains a common limitation of all current PRRSV‐MLVs, including commercial products based on HP‐PRRSV or VR2332 strains. Our candidate AHFY‐F140 carries stable dual deletions in NSP2 and characteristic mutations in GP5, which can serve as genetic tags and may reduce the fitness of recombinant viruses. However, the potential recombination risk associated with NSP2 truncation and the cross‐lineage protective efficacy of this candidate still require further evaluation. In subsequent studies, we will conduct coinfection experiments with AHFY‐F140 and representative field strains to systematically evaluate the recombination frequency.

Animal trials confirmed that the candidate‐modified live vaccine strain AHFY‐F140 has been successfully obtained. Compared with its parental virus AHFY‐F1, AHFY‐F140 showed significantly reduced virulence in piglets, as reflected by lower viral loads postinfection, an absence of obvious clinical signs, and no apparent gross pathological lesions in the lungs. Moreover, the AHFY‐F140 strain exhibited favorable immunogenicity and protective efficacy in piglets. Clinically, piglets immunized with AHFY‐F140 were effectively able to resist challenges with both the homologous AHFY‐F1 strain and the heterologous JXA1 strain (lineage 8). These immunized animals showed no PRRS‐related clinical signs, and their average daily weight gain was significantly higher than that of the challenge control group, indicating that AHFY‐F140 immunization prevented the acute symptoms caused by both HP‐PRRSV and NADC30‐like PRRSV infection.

At the immunology level, immunization with AHFY‐F140 elicited a strong humoral immune response and virus‐specific antibodies, but no significant neutralizing antibody response was detected during the trial period. Notably, viral loads in lung tissues of immunized piglets were ~100‐fold lower than those in controls at 21 dpc. These results collectively indicate that the immune response induced by AHFY‐F140 effectively restricts the extensive replication of HP‐PRRSV and NADC30‐like in target organs.

Pathological examinations further confirmed the protective effect. Lungs of piglets in the challenge mock group displayed typical PRRS lesions, including consolidation, hemorrhage, and extensive inflammatory cell infiltration. In sharp contrast, no gross or histopathological lesions were observed in the lungs of AHFY‐F140‐immunized animals. To date, the safety assessment in this study has been strictly limited to weaned piglets, and future research is needed to utilize the pregnant sow model to further evaluate the risks of vertical transmission and toxicity.

**Figure 8:**
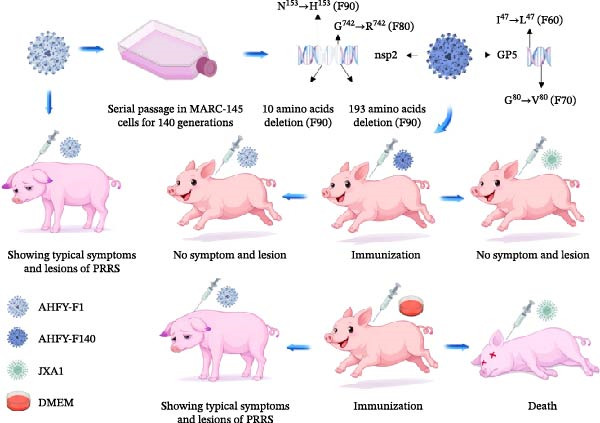
Schematic of AHFY attenuated strain preparation, pathogenicity evaluation, and challenge protection experiment.

In this study, we isolated a novel NADC30‐like PRRSV strain, AHFY, and generated an attenuated vaccine candidate, AHFY‐F140, via 140 serial passages in MARC‐145 cells. The resulting AHFY‐F140 strain harbors novel, stable large‐fragment dual deletions in the NSP2 virulence‐associated region as well as mutations in GP5. These molecular characteristics are analogous to those observed in several commercially available MLV strains (e.g., TJM‐F92 and RespPRRS MLV) and are accompanied by robust replicative capacity in MARC‐145 cells. Animal studies confirmed that AHFY‐F140 is safe in piglets and confers protective efficacy against challenge with both the homologous AHFY‐F1 strain and the heterologous JXA1 (HP‐PRRSV) strain. Collectively, these findings advance our understanding of PRRSV molecular evolution during cell culture attenuation and present AHFY‐F140 as a promising MLV candidate for controlling both NADC30‐like and HP‐PRRSV infections (Figure 8).

## Author Contributions

Conceptualization: Heng Zhang, Jiakai Zhao, and Yani Sun. Methodology: Heng Zhang, Jiakai Zhao, Yingru Ma, and Xiaohong Qi. Formal analysis: Heng Zhang, Chengxin Zhang, Ting Tang, Qianyi Zhu, and Hui Zhou. Investigation: Yani Sun and Qin Zhao. Writing of the original draft: Heng Zhang and Jiakai Zhao. Review and editing of the manuscript: Yani Sun and Qin Zhao. Supervision and funding acquisition: Heng Zhang and Qin Zhao.

## Funding

The study is funded by the Key R&D Program of Shandong Province, China (The Major Scientific and Technological Innovation Project, Grant 2023CXGC010705), the Key International Cooperation Project of National Natural Science Foundation of China (Grant W2511028), the Natural Science Foundation of China (Grant 32273041), and the Shaanxi Province Key Research and Development Program‐Foreign Cooperation Field (General Project) (Grant 2025GH‐YBXM‐070).

## Disclosure

All authors contributed to the manuscript, approved the submitted version, and have read and agreed to the published version of the manuscript.

## Ethics Statement

The animal study was reviewed and approved by the Animal Care and Ethics Committee of Shandong SINDER Technology Co., Ltd. (Approval Number 2024033001). All procedures were performed in accordance with the National Institutes of Health Guide for the Care and Use of Laboratory Animals, and every effort was made to minimize the suffering and number of animals used.

## Conflicts of Interest

The authors declare no conflicts of interest.

## Data Availability

The data that support the findings of this study are available upon reasonable request. The data are not publicly available due to ethical considerations.
